# Supplementary material for: A fault-intrusion-tolerant system and deadline-aware algorithm for scheduling scientific workflow in the cloud
Source: PeerJ Comput Sci. 2021 Nov 2;7:e747. doi: 10.7717/peerj-cs.747 (PMC8576554; doi:10.7717/peerj-cs.747)
Supplement: Supplemental Information 6 [file peerj-cs-07-747-s006.zip › Myproject1.1/docs/org/cloudbus/cloudsim/power/PowerVmAllocationPolicySimple.html]

PowerVmAllocationPolicySimple


JavaScript is disabled on your browser.


- Overview
- Package
- Class
- Use
- Tree
- Deprecated
- Index
- Help

- Prev Class
- Next Class

- Frames
- No Frames

- All Classes

- Summary:
- Nested |
- Field |
- Constr |
- Method

- Detail:
- Field |
- Constr |
- Method


org.cloudbus.cloudsim.power

## Class PowerVmAllocationPolicySimple

- java.lang.Object
- - org.cloudbus.cloudsim.VmAllocationPolicy
  - - org.cloudbus.cloudsim.power.PowerVmAllocationPolicyAbstract
    - - org.cloudbus.cloudsim.power.PowerVmAllocationPolicySimple

- ---

    

  ```
  public class PowerVmAllocationPolicySimple
  extends PowerVmAllocationPolicyAbstract
  ```

  This a simple class representing a simple VM allocation policy that does not perform any
  optimization of the VM allocation.
  If you are using any algorithms, policies or workload included in the power package, please cite
  the following paper:
  Anton Beloglazov, and Rajkumar Buyya, "Optimal Online Deterministic Algorithms and Adaptive
  Heuristics for Energy and Performance Efficient Dynamic Consolidation of Virtual Machines in
  Cloud Data Centers", Concurrency and Computation: Practice and Experience (CCPE), Volume 24,
  Issue 13, Pages: 1397-1420, John Wiley & Sons, Ltd, New York, USA, 2012

  Since:
  :   CloudSim Toolkit 3.0

- - ### Constructor Summary

    Constructors

    Constructor and Description

    `PowerVmAllocationPolicySimple(java.util.List<? extends Host> list)`

    Instantiates a new power vm allocation policy simple.
  - ### Method Summary

    Methods

    | Modifier and Type | Method and Description |
    | --- | --- |
    | `java.util.List<java.util.Map<java.lang.String,java.lang.Object>>` | `optimizeAllocation(java.util.List<? extends Vm> vmList)` Optimize allocation of the VMs according to current utilization. |

    - ### Methods inherited from class org.cloudbus.cloudsim.power.PowerVmAllocationPolicyAbstract

      `allocateHostForVm, allocateHostForVm, deallocateHostForVm, findHostForVm, getHost, getHost, getVmTable`
    - ### Methods inherited from class org.cloudbus.cloudsim.VmAllocationPolicy

      `getHostList, setHostList`
    - ### Methods inherited from class java.lang.Object

      `clone, equals, finalize, getClass, hashCode, notify, notifyAll, toString, wait, wait, wait`

- - ### Constructor Detail


    - #### PowerVmAllocationPolicySimple

      ```
      public PowerVmAllocationPolicySimple(java.util.List<? extends Host> list)
      ```

      Instantiates a new power vm allocation policy simple.

      Parameters:
      :   `list` - the list
  - ### Method Detail


    - #### optimizeAllocation

      ```
      public java.util.List<java.util.Map<java.lang.String,java.lang.Object>> optimizeAllocation(java.util.List<? extends Vm> vmList)
      ```

      **Description copied from class: `VmAllocationPolicy`**

      Optimize allocation of the VMs according to current utilization.

      **Specified by:**
      :   `optimizeAllocation` in class `VmAllocationPolicy`

      Parameters:
      :   `vmList` - the vm list

      Returns:
      :   the array list< hash map< string, object>>


- Overview
- Package
- Class
- Use
- Tree
- Deprecated
- Index
- Help

- Prev Class
- Next Class

- Frames
- No Frames

- All Classes

- Summary:
- Nested |
- Field |
- Constr |
- Method

- Detail:
- Field |
- Constr |
- Method
